# Integrated metabolomics and transcriptome analysis on flavonoid biosynthesis in flowers of safflower (*Carthamus tinctorius* L.) during colour-transition

**DOI:** 10.7717/peerj.13591

**Published:** 2022-06-22

**Authors:** Chaoxiang Ren, Chao Chen, Shuai Dong, Rui Wang, Bin Xian, Tianlei Liu, Ziqing Xi, Jin Pei, Jiang Chen

**Affiliations:** 1 State Key Laboratory of Southwestern Chinese Medicine Resources, Chengdu, Sichuan, China; 2 College of Pharmacy, Chengdu University of Traditional Chinese Medicine, Chengdu, Sichuan, China; 3 The State Bank of Chinese Drug Germplam Resources, Chengdu University of Traditional Chinese Medicine, Chengdu, Sichuan, China; 4 College of Medical Technology, Chengdu University of Traditional Chinese Medicine, Chengdu, Sichuan, China

**Keywords:** Flavonoid glycosides biosythesis, Glycosyltransferases, Colour-transition, Metabolomics, Transcriptome, Safflower

## Abstract

**Background:**

Safflower (*Carthamus tinctorius* L.), well known for its flower, is widely used as a dye and traditional Chinese medicine. Flavonoids, especially flavonoid glycosides, are the main pigments and active components. However, their biosynthesis is largely unknown. Interestingly, the colour of flowers in safflower changed from yellow to red during flower development, while much of the gene and chemical bases during colour transition are unclear.

**Methods:**

In this research, widely targeted metabolomics and transcriptomics were used to elucidate the changes in flavonoid biosynthesis from the gene and chemical points of view in flowers of safflower during colour transition. The screening of differential metabolites depended on fold change and variable importance in project (VIP) value. Differential expressed genes (DEGs) were screened by DESeq2 method. RT-PCR was used to analyse relative expressions of DEGs.

**Results:**

A total of 212 flavonoid metabolites, including hydroxysafflor yellow A, carthamin and anthocyanins, were detected and showed a large difference. The candidate genes of glycosyltransferases and flavonoid hydroxylase that might participate in flavonoid glycoside biosynthesis were screened. Ten candidate genes were screened. Through integrated metabolomics and transcriptome analysis, a uridine diphosphate glucose glycosyltransferase gene, *CtUGT9* showed a significant correlation with flavonoid glycosides in safflower. In addition, expression analysis showed that *CtUGT9* was mainly expressed in the middle development of flowers and was significantly upregulated under MeJA treatment. Our results indicated that *CtUGT9* might play an important role in flavonoid glycoside biosynthesis during colour-transition in safflower.

## Introduction

Safflower (*Carthamus tinctorius* L.), also known as Hong Hua in China, is a member of the family Compositae or Asteraceae (Ashri et al., 1975). It is an annual, self-compatible, diploid (2n = 2x = 24) crop with a long cultivation history, and it is believed to have been domesticated approximately 4,000 years ago in the Fertile Crescent and has various centres of origins (Knowles, 1969). Safflower has been cultivated and used in China for more than 2,000 years, as recorded in the compendium of Materia Medica. Zhang Qian introduced safflower to China during the Han Dynasty on a diplomatic mission to the Western Regions (*via* the Silk Road). Safflower is known for its flowers and is used as dyes, cosmetics, and food additives worldwide (Mohammadi & Tavakoli, 2015; Azami et al., 2019). At the same time, in China and other Southeast Asian countries, dried tubular flowers are used in traditional Chinese medicine to improve cerebral blood flow and to treat various ailments, such as cerebrovascular and cardiovascular diseases (Jin et al., 2008), hypertension (Asgarpanah & Kazemivash, 2013), and coronary heart disease (Zhou et al., 2018; Chinese Pharmacopoeia Commission, 2015). Flavonoids are the main metabolite in safflower. Flavonoid glycosides (such as hydroxysafflor yellow A (HSYA)) are not only pigments of safflower (Kazuma et al., 2000; Wang et al., 2021a) but also the active component (Yang et al., 2009; Ao, Feng & Peng, 2018; Bai et al., 2020). However, their biosynthesis is largely unknown.

**Figure 1:**
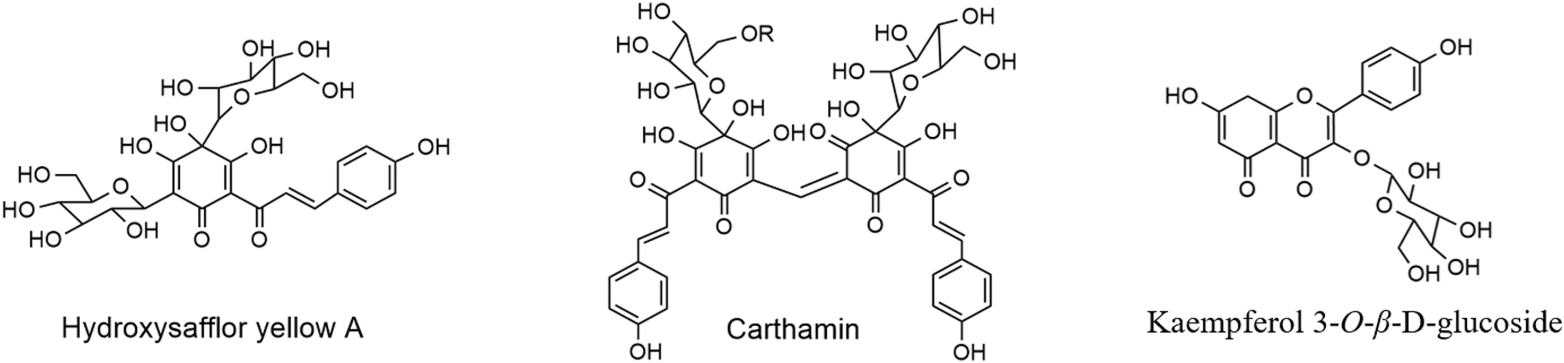
Structure of three flavonoid glycosides in safflower. They are Hydroxysafflor yellow A (HSYA), carthamin and kaempferol-3-*O*-*β*-D-glucoside from left to right.

Currently, the core flavonoid biosynthetic pathway is well understood (Grotewold, 2006; Zhang, Butelli & Martin, 2014; Zhao & Tao, 2015). In safflower, according to a reported paper (Yue et al., 2013; Pu et al., 2021), there are mainly several flavonoid glycosides in safflower, such as HSYA, carthamin, and kaempferol-3-*O*-*β*-D-glucoside. According to their structure (Fig. 1), two kinds of glycosyltransferases might take part in their biosynthesis: *O*-glycosyltransferases (OGTs) (for the biosynthesis of kaempferol-3-*O*-*β*-D-glucoside) and *C*-glycosyltransferases (CGTs) (for the biosynthesis of HSYAs). In addition, according to the structure of HSYA, flavonoid hydroxylase, especially flavonoid four hydroxylase (Cytochrome P450, CYPs), might participate in its biosynthesis. In addition, there are many glycosyltransferases in other plants, such as *C*-glucosyl flavonoids in citrus plants (Ito et al., 2017), *Glycyrrhiza glabra* (Zhang et al., 2020), *O*-glucosyl flavonoids in *Medicago truncatula* (Luzia et al., 2007) and flavonoid hydroxylase in Scutellaria baicalensis (Zhao et al., 2018). Their work provides a reference for us to screen candidate genes involved in flavonoid glycoside biosynthesis in safflower.

Note that the flowers of safflower change their colour from yellow (Y) to red (R) during flowering (Ren et al., 2019; Pu et al., 2021). However, much of the gene and chemical bases during colour transition are still unclear. There are some reports on the chemical composition of safflower, while most research uses HPLC coupled with DAD, HPLC coupled with MS, or even UHPLC-Q-TOF-MS to analyze the components of safflower (Kazuma et al., 2000; Yue et al., 2013; Kim et al., 2020; Pu et al., 2021). However, most of these reports have some shortcomings, such as a small number of compounds. In recent years, widely targeted metabolomics based on UHPLC–ESI–MS/MS has become very popular in the analysis and identification of compounds due to its advantages of high throughput, fast separation, high sensitivity, and wide coverage. At present, this method has been widely applied in plant metabolite analysis in rice (Chen et al., 2013), maize (Wen et al., 2014), tomato (Zhu et al., 2018; Yang et al., 2021), potato (Cho et al., 2016), and other plants (Wang et al., 2021b; Xie et al., 2020). We believe that this method can be used to detect the chemical changes during flower colour transition in safflower.

Recently, analysis of the metabolomics combined with the transcriptome had widely used in revealing the mechanisms of many important physiological processes (Scossa, Alseekh & Fernie, 2021; Xia et al., 2021; Zou et al., 2021). In research on *Lonicera japonica* Thunb, integrated metabolomics and transcriptome analysis of pigment accumulation in *Lonicera japonica* flower petals during colour transition revealed changes in key pigments and related biosynthesis genes associated with petal colour transitions (Xia et al., 2021). Besides, in our previously research, through integrated metabolomics and transcriptome methods, we analyze the molecular mechanism of flavonoid biosynthesis under MeJA treatment in safflower (Chen et al., 2020). Therefore, we believe that through integrated metabolomics and transcriptome analysis on flavonoid biosynthesis in safflower flowers during colour transition, some genes associated with flower colour transitions can be elucidated.

In this research, widely targeted metabolomics and transcriptomics were used to elucidate the changes from gene and chemical points of view in flavonoid biosynthesis in safflower flowers during colour transition. Expression profile of the genes that were significantly correlated with flavonoid glycosides in safflower were analysed. We aimed to elucidate the colour change from a chemical point of view and to screen candidate genes that participate in flavonoid glycoside biosynthesis through integrated metabolomics and transcriptome analysis of safflower flowers during colour transition.

## Materials and Methods

### Plant materials and sampling

**Figure 2:**
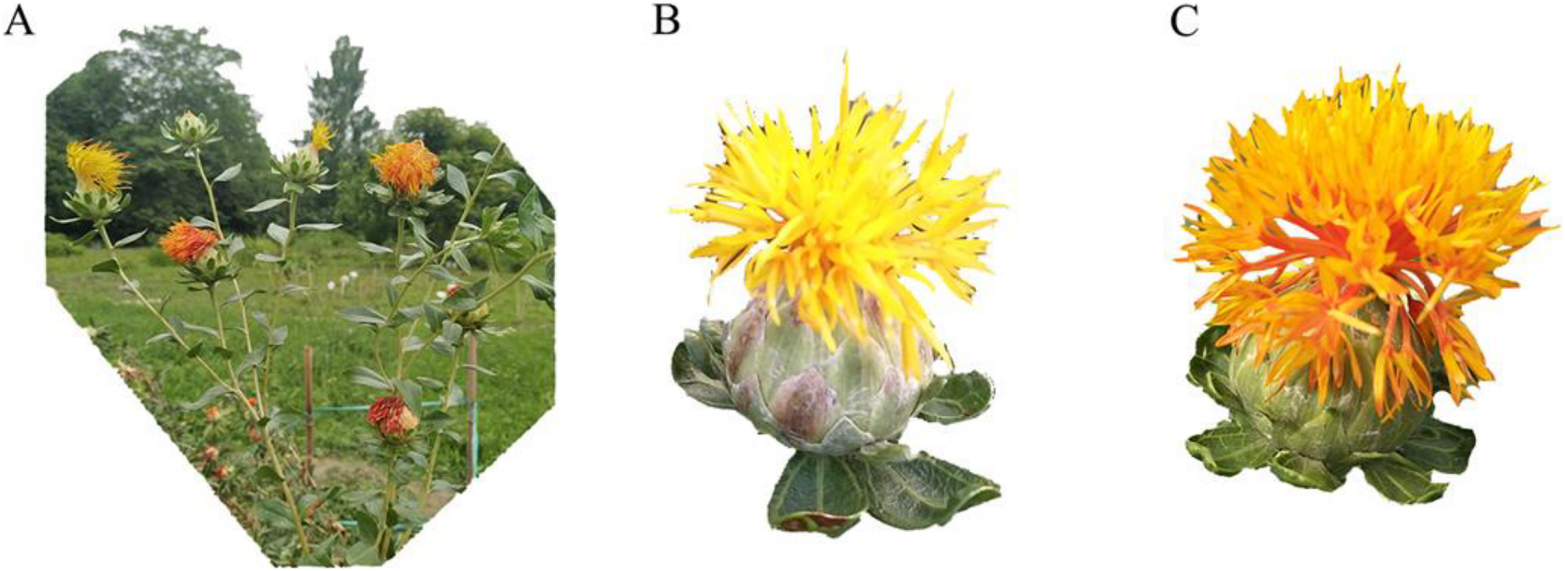
The colour transition of flowers in safflower. (A) Colour transition in flower at different development of flower in the same plant. (B) The flower phenotype at Day 2. (C) The flower phenotype at Day 4.

Safflower, “Chuanhonghua No. 1”, was used in this experiment. It was selected and bred by the Industrial Crop Research Institute, Sichuan Academy of Agricultural Sciences, and presented by RenchuanYao to our lab. In this research, it was cultivated in a medicinal botanical garden at the Wenjiang campus of Chengdu University of Traditional Chinese Medicine in 2019. In our previous research, we recorded the development of safflower flowers, and the flowering period time of safflower can last 7 days, where the colour of flowers transitions from yellow (Y) to red (R) from Day 2 to Day 4. Thus, we chose the flowers of Day 2 and Day 4 as the samples in our study (Fig. 2). Samples of flowers were collected, frozen immediately in liquid nitrogen and stored in a freezer at −80 °C. For RNA sequencing, five inflorescences of safflower at the same flowering stage were mixed as one sample. For metabolism analysis, 10 inflorescences of safflower plants at the same flowering stage were mixed as one sample. Three biological replicates were performed for transcriptomics metabolomics analysis.

### Sample preparation and extraction for metabolomic analysis

This sample preparation and extraction were mainly consistent with our previous research (Chen et al., 2020). The freeze-dried flowers were crushed using a mixer mill (MM 400; Retsch, Haan, Germany) with a zirconia bead for 1.5 min at 30 Hz. A total of 100 mg of powder was weighed and extracted overnight at 4 °C with 1.0 mL of 70% aqueous methanol and then centrifuged at 10,000 g for 10 min. The extracts were absorbed (CNWBOND Carbon-GCB SPE Cartridge, 250 mg, 3 mL; ANPEL, Shanghai, China, www.anpel.com.cn/cnw) and filtered (SCAA-104, 0.22 μm pore size; ANPEL, Shanghai, China) before LC–MS analysis. The UHPLC and mass spectrometry conditions also followed those of previous research (Chen et al., 2020).

### Qualitative and quantitative analysis of metabolites

Flavonoid identification and quantification in our study were performed according to a previous report (Chen et al., 2020). Besides, in our previous research, carthamin was not included. This time, we used the standard sample of carthamin to build a library and added them to the homemade database. The metabolites of the samples were qualitatively and quantitatively analysed by mass spectrometry. The integrated area peak of each compound was used for principal component analysis (PCA) and orthogonal partial least squares-discriminant analysis (OPLS-DA).

### Differential metabolite analysis

The fold change and variable importance in project (VIP) value of the OPLS-DA model were combined to screen differential metabolites as described in our previous research (Chen et al., 2020). PCA was performed by using R software built-in functions (www.r-project.org/). For the parameter, scale = True. After conversion of the original data by log2, the data were centralized (Mean Centering) and analyzed. The screening criteria were as follows: 1. Metabolites must exhibit fold changes ≥2 and fold changes ≤0.5. If a metabolite in the experimental group exhibited a fold change more than two times or less than 0.5 times that of the metabolite in the control group, the difference was considered significant 2. If there was biological duplication in the sample group, the metabolites of VIP ≥1 were selected based on the above. The VIP value indicates the influence intensity of the difference between groups of corresponding metabolites in the classification and discrimination of each group of samples in the model.

### RNA sequencing and annotation

RNA isolation and sequencing were performed as previously described (Love, Wolfgang & Simon, 2014). The samples were ground on dry ice, and total RNA was prepared by using TRIzol reagent (Invitrogen, Carlsbad, CA, USA). To remove DNA, an aliquot of total RNA was treated with DNase (Takara, Dalian, China). RNA was determined by using a NanoDrop 2000 spectrophotometer (NanoDrop, Wilmington, DE, USA) and an Agilent 2100 bioanalyzer (Agilent, Santa Clara, CA, USA). mRNA was isolated from total RNA using magnetic beads with oligo (dT) primers; cDNA was synthesized by using a cDNA synthesis kit (Takara, Dalian, China).

The library preparations were sequenced on an Illumina HiSeq platform with three biological replicates in each group. In this project, Trinity v2.0.6 (Grabherr et al., 2011) was used to splice the filtered high-quality sequencing data to obtain the transcriptome, which was used as the reference sequence for subsequent differential expression analysis. After the transcriptome was assembled, clean reads were compared with the transcriptome. The average sequencing depth (counts * 150/gene_len) was about 424.

A power analysis was performed by RNASeqpower in R package (Hart et al., 2013). The statistical power of this experimental design, calculated in RNASeqpower was 0.9210878 (depth = 424, *n* = 3, CV = 0.24, effect = 2, alpha = 0.05).

The UniGene sequences obtained were annotated through six databases: KEGG, GO, NR, Swiss-Prot, trEMBL and KOG. The whole set of transcript data can be found in the National Center for Biotechnology Information (NCBI) database (BioProject ID: PRJNA774916).

### Screening of differential genes

The screening of differential expressed gene was mainly consistent with our previous research (Wang et al., 2021a). The gene expression levels were calculated through RSEM (RNAseq by Expectation-Maximization) v1.2.12 (Li & Colin, 2011). DESeq2 (Love, Wolfgang & Simon, 2014) was used for differential expression analysis. Read counts of genes are the expected count outputs calculated using RSEM. After discrepancy analysis, multiple hypothesis tests are needed to correct the hypothesis test probability (*P* value) with the Benjamini-Hochberg procedure to obtain the false discovery rate (FDR) when |log2fold change| ≥ 1 and FDR < 0.05.

### Expression profile in the development of different flower tissues

According to previous research (Ren et al., 2019), the development of safflower can be listed for 7 days. However, in the last 2 days, the flowers are almost dry. Thus, we chose the beginning of the first 5 days to check gene expression. In addition, the root, stem and leaf at anthesis were used to detect gene expression in different tissues. RT–PCR was used to measure their expression, as previously described (Ren et al., 2019). Total RNA was isolated using an RNA extraction kit (Invitrogen, CA, USA), and reverse transcription was carried out using the Prime Script Reagent Kit (Takara, Dalian, China). The primers used to amplify the screened gene *CtUGT9* by real-time PCR were designed by Primer 5.0, with the sequences ACTCCAAAGGCTTCTCAAT (Forward) and GGTGTAGTGTTAAAGGGTAAAT (Reverse), and parts of the safflower 28S coding region were used as internal reference genes, with the sequences GGGTCCTTTCACGTTTCTGA (Forward) and GGCCTGACTTATCGGTAGCA (Reverse). RT–PCR was performed using SYBR Premix Ex Taq II (Takara, Shiga, Japan) with three replicates, and the cycling conditions were set according to the manual. The Bio–Rad CFX96 real-time system (Hercules, CA, USA) was used in this experiment.

### MeJA treatment of the flower

The MeJA treatment of the flowers was mainly performed according to our previous research (Chen et al., 2020). A 100 μM MeJA (Sigma–Aldrich, St. Gallen, Switzerland) was sprayed onto healthy safflower flowers 3 days after anthesis (DAA). The flowers sprayed with the same solution without MeJA were used as the control group. The flowers were then enclosed in clear plastic bags. After treatment for 6 h, flowers were collected, immediately frozen in liquid nitrogen for further analysis.

## Results

### Metabolic profiling and differential flavonoid analysis in flowers of safflower during colour transition

The flavonoids in flowers of safflower during colour transition from yellow to red were detected based on UHPLC ESI-MS/MS (including HSYA and carthamin). A total of 212 flavonoids were detected, including 64 flavones, 41 flavonols, 40 flavone C-glycosides, 22 flavonones, 10 isoflavones, 10 catechin derivatives, 19 anthocyanins, two quinone chalcones, two flavonolignans, one alkaloid, and one proanthocyanidin (Table S1).

**Figure 3:**
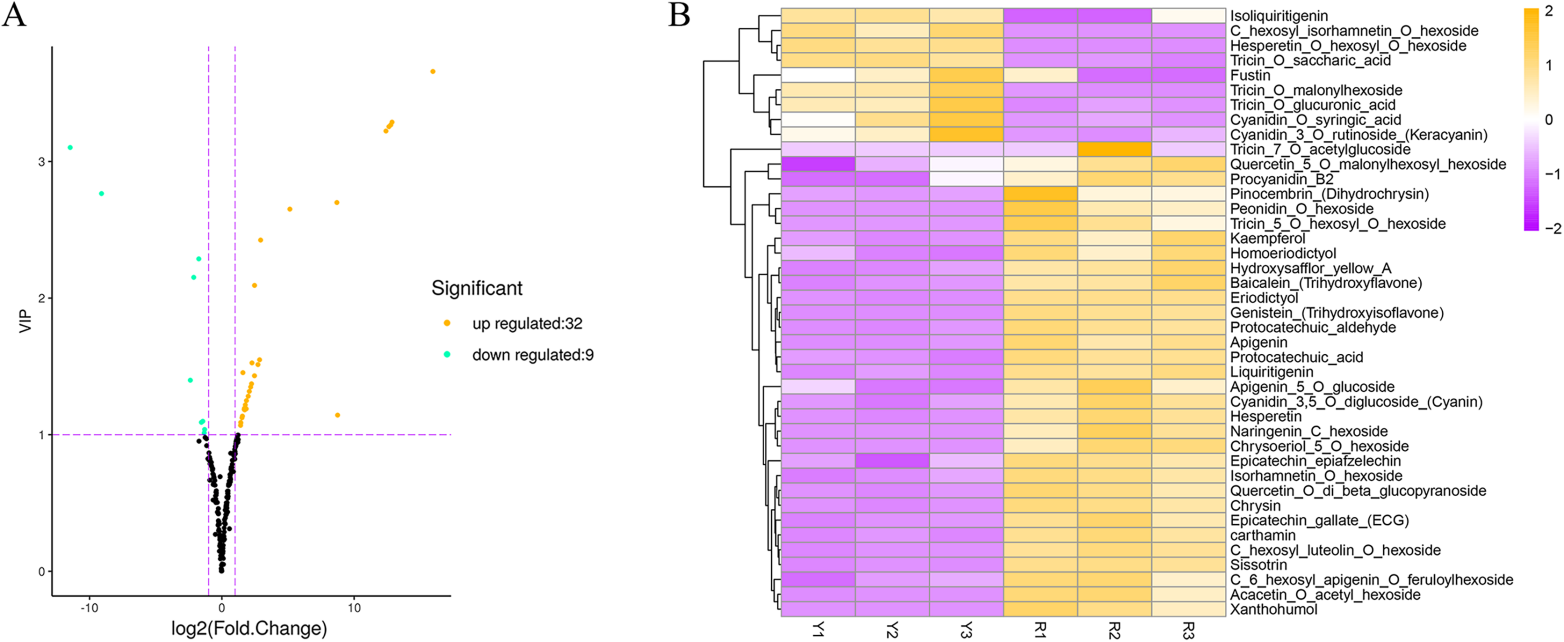
Differential flavonoid analysis in flowers of safflower during colour transition. (A) Volcanic map of differential flavonoids, red dot means the up-regulated flavonoid, while green dot means the down-regulated flavonoid. (B) Heatmap of the differential flavonoids.

PCA shows that the two samples (Y and R) can be clearly clarified based on flavonoids (Fig. S1). The fold change and VIP value of the OPLS-DA model were combined to screen differential flavonoids. There were 41 significantly differential flavonoids in the flowers of safflower during the colour transition from Y to R. Among them, 32 flavonoids were upregulated, and nine flavonoids were downregulated (Fig. 3A). A heatmap of the differential flavonoids is drawn (Fig. 3B). It shows that 4 flavone *C*-glycosides are differently regulated, among which three flavone *C*-glycosides are significantly upregulated from Y to R. It also shows that four anthocyanins are differently regulated, and two anthocyanins (peonidin *O*-hexoside and cyanidin 3,5-*O*-diglucoside) are significantly upregulated from Y to R. All the details of the differential flavonoids can be viewed in Table S2.

### Transcriptome sequencing and differential transcript analysis in flowers of safflower during colour transition

The transcriptome of flowers during colour transition was sequenced: 47.49 G clean bases were obtained, including 320,594,102 raw reads and 318,575,696 clean reads. The Q30 value was above 93.3%. The details can be viewed in Table S3. Trinity (Grabherr et al., 2011) was used to splice the filtered high-quality sequencing data to obtain the transcriptome as a reference sequence for subsequent differential expression analysis. A total of 437,060 transcripts and 270,890 UniGenes were extracted (Table S4). Six databases (KEGG, GO, NR, Swiss-Prot, trEMBL and KOG) were used for protein annotation, and 90,961 genes were annotated by at least one database (Table S5).

**Figure 4:**
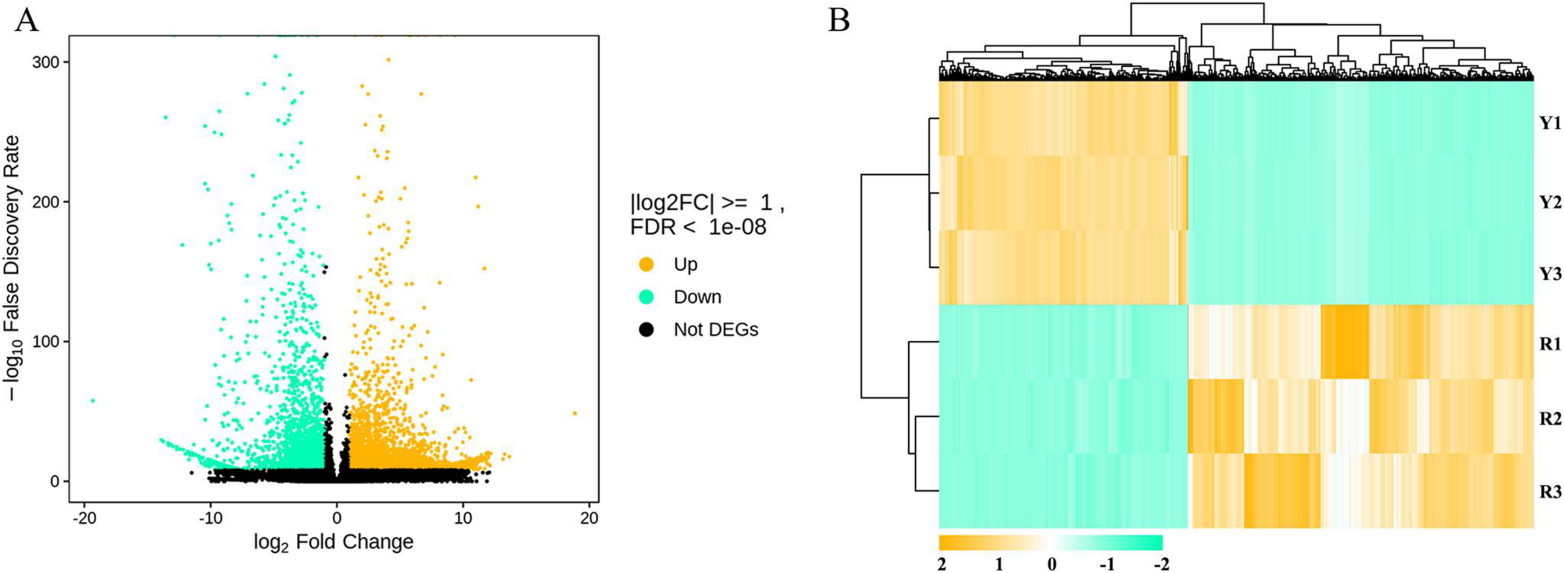
Differential transcript analysis in flowers of safflower during colour transition. (A) Volcanic map of differently expressed genes, red dot means the up-regulated genes, while green dot means the down-regulated genes. (B) Heatmap of differentially expressed genes. Cluster analysis was performed according to gene expression levels.

The differentially expressed genes were analyzed with DESeq2 (Love, Wolfgang & Simon, 2014). A total of 4,820 genes were differentially expressed in the flowers of safflower during colour transition, among which 2,018 genes were downregulated and 2,802 genes were upregulated (Fig. 4). The details can be viewed in Table S6. The differentially expressed genes were annotated through the KEGG database. The differential genes mainly participated in metabolic pathways (648), biosynthesis of secondary metabolites (377), ribosomes (276), and MAPK signalling (171). Among them, 67 genes participated in phenylpropanoid biosynthesis, including 18 genes that participated in flavonoid biosynthesis (KO00941), three genes that participated in anthocyanin biosynthesis (KO00942), and two genes that participated in flavone and flavonol biosynthesis (KO00944). The details can be viewed in Table S7.

### Screening and expression analysis of glycosyltransferases and flavonoid hydroxylase participating in flavonoid glycoside biosynthesis

It can be inferred from the structure of the flavonoid glycosides in safflower (Yue et al., 2013; Pu et al., 2021) (Fig. 1) that two kinds of UGT might participate in flavonoid glycosides, *O*-glycosyltransferases (OGTs) and *C*-glycosyltransferases (CGTs). As all of the UGT family members have the domain PF00201 (UDP-glucoronosyl and UDP-glucosyl transferase domains), the UGT gene sequences were first extracted from the transcriptome of safflower flowers based on PF00201. A total of 79 genes were screened. Phylogenetic analysis was performed with the reported UGTs (Luzia et al., 2007; Ito et al., 2017; Zhang et al., 2020), including 11 CGTs and 6 OGTs. Nine candidate UGTs were screened, including 2 CGTs and 7 OGTs, which were named *CtUGT1*-*CtUGT9* (Fig. S2). The genes used for BLAST in the research are listed in Table S8. In addition, according to the structure of HSYA, flavonoid hydroxylases, isoforms such as CYP82D2 in *Scutellaria baicalensis* (Zhao et al., 2018), might participate in flavonoid hydroxylase in safflower. All flavonoid hydroxylases contain the domain PF00067 (cytochrome P450 domain). Therefore, all flavonoid hydroxylase gene sequences were extracted from the transcriptome of flowers of safflower on PF00067. A total of 271 genes were screened. Then, phylogenetic analysis was performed with CYP82D2. One homologue of the CYP82D gene was screened and named *CtCYP1* (Fig. S3). All the sequences of the screened genes are listed in Table S9.

### Integrated metabolomics and transcriptome analysis in flowers of safflower during colour transition

**Figure 5:**
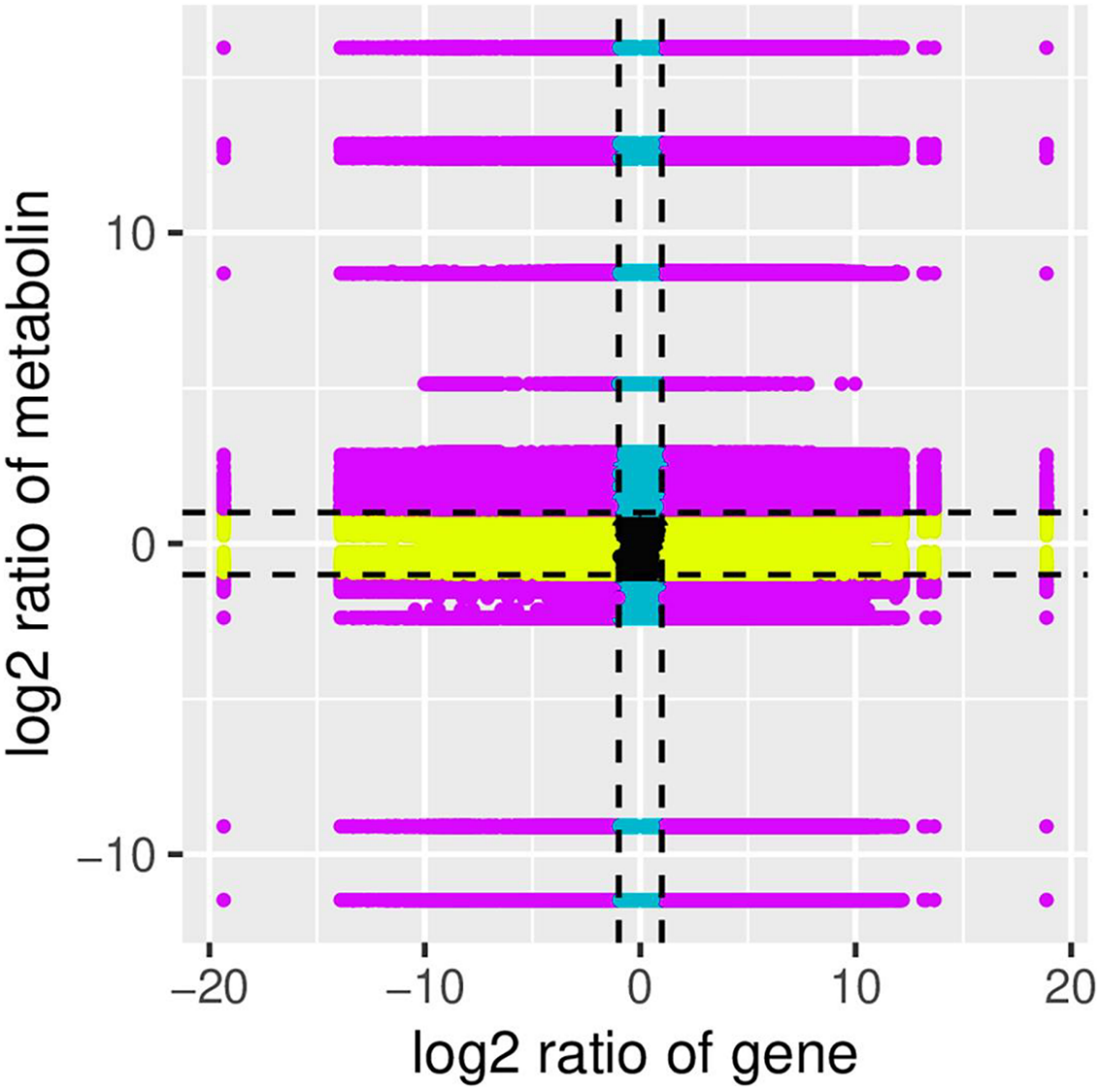
The nine quadrants constructed based on the pearson correlation coefficient. Black dashed lines was used to divide the figure into 1–9 quadrants. Five means that genes and flavonoids are not differently expressed. Three and seven mean differential expression patterns of genes and flavonoids are consistent, and genes and flavonoids that are positively correlated in the two quadrants. Metabolite changes may be positively regulated by genes. One, two and four mean that the expression abundance of metabolite is higher than that of genes. Genes and flavonoids are negatively correlated to each other, which means that flavonoids are up-regulated, while genes are unchanged or down-regulated. Six, eight and nine mean that the expression abundance of metabolite is lower than that of genes. Genes are negatively correlated to flavonoids. When genes are up-regulated, flavonoids remain unchanged or down-regulated.

**Figure 6:**
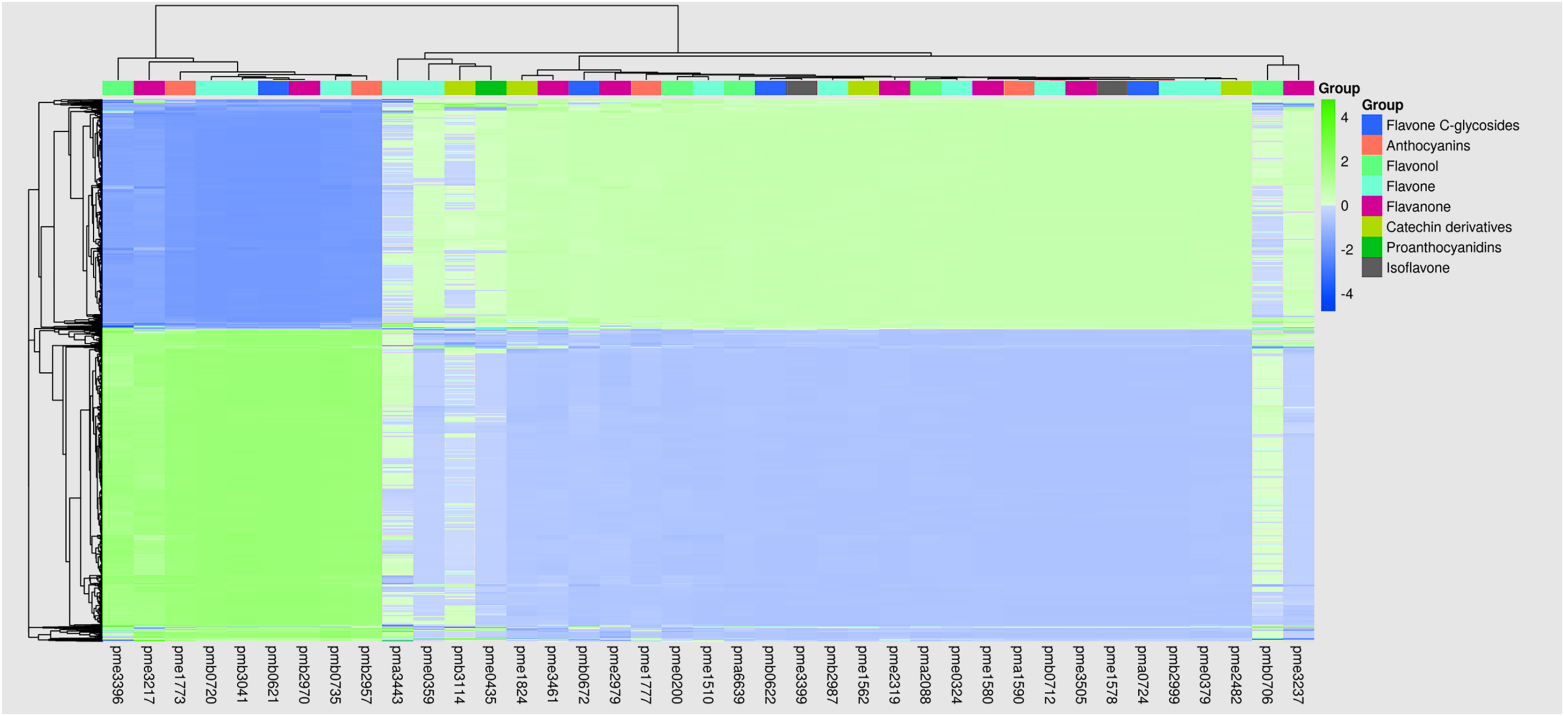
The clustering heat map of genes and flavonoids. The result is showed based on the pearson correlation coefficient greater than 0.8.

In this study, nine quadrants were constructed to perform the integrated metabolomics and transcriptome analyses. Correlation analysis of the genes and metabolites detected in flowers of safflower during colour transition was performed using the cor program in R to calculate the Pearson correlation coefficients between the genes and metabolites. The nine quadrants were constructed based on the Pearson correlation coefficient (Fig. 5). The results with Pearson correlation coefficients greater than 0.8 were selected, and the clustering heatmap based on the correlation coefficient is shown in Fig. 6. The nine genes screened through phylogenetic analysis were analysed, and the results showed that only the *CtUGT9* gene (TRINITY_DN101065_c9_g1) was positively correlated (PPC above 90%) to 25 flavonoids, including one flavonoid glycoside. Details are shown in Table S10.

### Expression profile of CtUGT9 and its response to MeJA treatment

**Figure 7:**
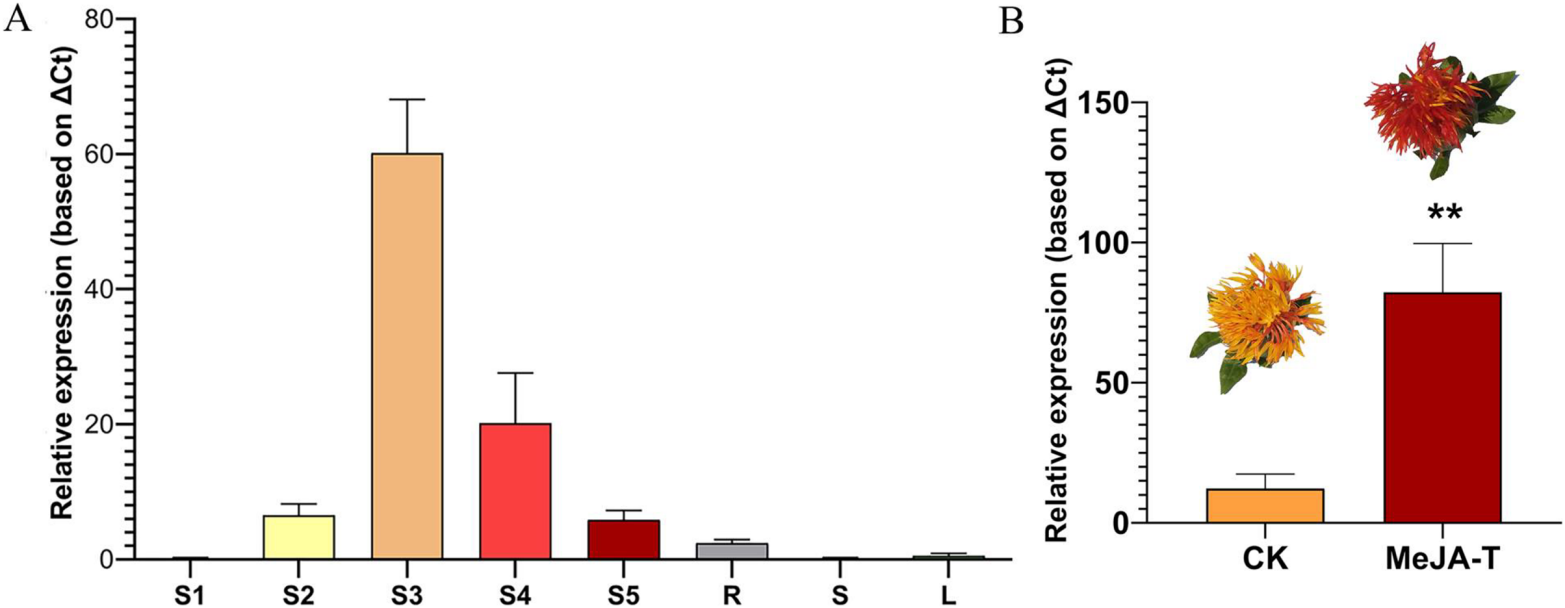
The expression profile of the *CtUGT9* and its response to MeJA treatment. (A) The expression profile of the *CtUGT9*. S1–S5 mean five different development stages of flower in safflower. R means Root, S means Stem, L mean leaf. (B) *CtUGT9* response to MeJA treatment. CK means untreatment of MeJA. MeJA-T mean the treatment of MeJA. Two asterisks (**) mean significance at 0.01%. The flower phenotypes of untreatment and treatment of MeJA on safflower were shown in the figure.

Integrated metabolomics and transcriptome analysis in flowers of safflower during colour transition indicated that *CtUGT9* is significantly correlated with many flavonoids, including one flavonoid glycoside, indicating that it might play an important role in the biosynthesis of flavonoid glycosides. Gene expression is closely related to its function; thus, we further detected the expression profile of *CtUGT9*. Its expression in different tissues (root, stem, leaf and flower tissue) and in the development of flowers was detected. The results showed that *CtUGT9* was mainly expressed in flowers, and along with the development of flowers, the expression of CtUGT9 showed a trend of rising first and then decreasing, with the highest expression at Day 3 of flowering (Fig. 7A).

Our previous study reported that flavonoids of safflower, including flavonoid glycosides, can be upregulated after MeJA treatment (Chen et al., 2020). Therefore, we speculate that *CtUGT9* should also be upregulated by MeJA. Thus, its expression under MeJA treatment was detected. The results showed that there was a significant increase after MeJA treatment (*P* = 0.01) (Fig. 7B). Overall, the results indicated that *CtUGT9* might play an important role in the biosynthesis of flavonoids and flavonoid glycosides in the flowers of safflower.

## Discussion

The flowers of safflower have multipurpose usage, not only in dyes and cosmetics but also in traditional Chinese medicine. There are two well-known pigments in safflower: yellow pigments, such as HSYA, which is also an active component in traditional Chinese medicine, and red pigments, such as carthamin (Kazuma et al., 2000; Pu et al., 2021). Our results showed that during the colour transition of safflower flowers, HSYA and carthamin showed a substantial difference, with a significant increase from Y to R, which is the same as a previous report (Pu et al., 2021). However, anthocyanins were also detected and showed a significant difference during color transition in safflower. Four anthocyanins were differentially regulated, among which 2 anthocyanins (peonidin *O*-hexoside and cyanidin 3,5-*O*-diglucoside) were significantly upregulated during the colour transition from Y to R. It is possible that anthocyanins might play a role in the colour transition of safflower.

A total of 4,820 genes were differentially expressed in the flowers of safflower during colour transition. Among these genes, 67 participate in phenylpropanoid biosynthesis, including 18 genes that participate in flavonoid biosynthesis (KO00941), three genes that participate in anthocyanin biosynthesis (KO00942), and two genes that participate in flavone and flavonol biosynthesis (KO00944). We analysed gene expression in the KEGG map (Fig. S4). Most genes downstream of the flavonoid biosynthesis pathway were downregulated during the colour transition of flowers. Only several genes upstream of the flavonoid biosynthesis pathway, such as *HCT* and *CHI*, were unregulated during colour transition. One possible reason is that the flavonoid components in safflower, especially flavonoid glycosides, branch upstream from the flavonoid biosynthesis pathway, which is not the same as the known pathway of flavonoid biosynthesis. In our previous report, we also found that after treatment with MeJA, the contents of flavonoid glycosides, especially HSYA components, increased, but gene expression downstream of the flavonoid biosynthesis pathway decreased (Chen et al., 2020). These results also reveal that the flavonoid biosynthesis pathway in safflower shows a substantial difference from the known flavonoid biosynthesis pathway in model plants, such as *Arabidopsis thaliana*.

In our experiment, 10 genes were screened as candidate genes that might participate in flavonoid glycoside biosynthesis in safflower, including *CtCYP1* and *CtUGT1*-*CtUGT9*. However, only *CtUGT9* showed a significant correlation with most flavonoids, including flavonoid glycosides. All the other 10 candidate genes were detected in our experiment, and the results showed that only *UGT9* was highly expressed in safflower and had a substantial change during the colour transition of flowers compared with the other nine genes (Fig. S5).

Flavonoid glycosides, the main components of the pigment in safflower, are mainly detected in flowers (Kazuma et al., 2000; Kim et al., 2020; Wang et al., 2021a; Pu et al., 2021). Gene expression is closely related to its function. We further detected the expression of *CtUGT9*, and the results showed that it was mainly expressed in flowers. During flower development, the expression of *CtUGT9* showed a trend of rising first and then decreasing (Fig. 7A). In addition, *CtUGT9* showed a strong response to MeJA treatment. The expression analysis indicated that *CtUGT9* might participate in flavonoid glycoside biosynthesis (Fig. 7B).

In our research, we planned to find CGTs and CYPs that might participate in flavonoid biosynthesis. However, the screened CYPs or CGTs did not seem to participate in flavonoid glycoside biosynthesis in safflower. The UGTs in safflower may differ from the reported UGTs. From the reported UGTs (Ito et al., 2017; Luzia et al., 2007; Zhang et al., 2020), most of the substrate contains flavonoids; however, most of the *C*-glycosyl in safflower is synthesized based on chalcone (Fig. 1). In addition, for HSYA, there is a −OH group at the four position. Therefore, in the future, to identify candidate genes that participate in flavonoid glycoside biosynthesis in safflower, especially flavonoid *C*-glycoside biosynthesis, some other methods might be used, such as GWAS (Chen et al., 2014) or mGWAS (Fang & Luo, 2019).

## Conclusions

Safflower (*Carthamus tinctorius* L) is widely used as a dye and traditional Chinese medicine. Flavonoids, especially flavonoid glycosides, are pigments and active components in safflower. Interestingly, the colour of safflower flowers changes from yellow to red during development. In this research, widely targeted metabolomics and transcriptomics were adapted to elucidate the changes from a gene and chemical point of view in flavonoid biosynthesis in flowers of safflower during colour transition. A total of 212 differential flavonoid metabolites and 4,820 differentially expressed genes were detected in the flowers of safflower during colour transition. Expression profiles of the genes that were significantly correlated with flavonoid glycosides in safflower were analysed. We found that *CtUGT9* was significantly associated with flavonoids and flavonoid glycosides. It was mainly expressed in flowers and showed a trend of rising first and then decreasing, with the highest expression at Day 3 of flowering. In addition, *CtUGT9* was significantly upregulated by MeJA treatment. Our results indicated that *CtUGT9* played an important role in flavonoid glycoside biosynthesis, which might affect the flower colour transition in safflower.

## Supplemental Information

10.7717/peerj.13591/supp-1 Supplemental Information 1 The flavonoids detected in this study.

10.7717/peerj.13591/supp-2 Supplemental Information 2 The details of the differential flavonoids.

10.7717/peerj.13591/supp-3 Supplemental Information 3 The details of the transcriptome data of flower during during colour transition.

10.7717/peerj.13591/supp-4 Supplemental Information 4 The details of transcripts and unigenes of flower during during colour transition.

10.7717/peerj.13591/supp-5 Supplemental Information 5 Gene annotations the six databases (KEGG, GO, NR, Swiss-Prot, trEMBL and KOG).

10.7717/peerj.13591/supp-6 Supplemental Information 6 Details of the differentially expressed genes in flowers of safflower during colour transition.

10.7717/peerj.13591/supp-7 Supplemental Information 7 KEGG annotation for the differential genes.

10.7717/peerj.13591/supp-8 Supplemental Information 8 Details of the reported UGT genes used in the phylogenetic analysis.

10.7717/peerj.13591/supp-9 Supplemental Information 9 Details of the candidate genes in this study.

10.7717/peerj.13591/supp-10 Supplemental Information 10 Details of *CtUGT9* gene correlated to the flavonoids.

10.7717/peerj.13591/supp-11 Supplemental Information 11 Principal component analysis of the sample based on flavonoids.

10.7717/peerj.13591/supp-12 Supplemental Information 12 Phylogenetic analysis of UGTs in safflower with the reported UGTs. The candidate genes are shown in red font.

10.7717/peerj.13591/supp-13 Supplemental Information 13 Phylogenetic analysis of flavonoid hydroxylase genes (CYPs) in safflower with the reported *CYP82D2*. The candidate genes are shown in red font.

10.7717/peerj.13591/supp-14 Supplemental Information 14 KEGG analysis of differential expressed genes involved in flavonoid biosynthesis.

10.7717/peerj.13591/supp-15 Supplemental Information 15 Expression analysis of candidates genes.

10.7717/peerj.13591/supp-16 Supplemental Information 16 Raw data for qRT-PCR in Figure 7.

## Additional Information and Declarations

### Data Availability

The following information was supplied regarding data availability: The transcriptome data is available at NCBI: PRJNA774916.
