# Supplementary figures and images for: Integrated metabolomics and transcriptome analysis on flavonoid biosynthesis in flowers of safflower (Carthamus tinctorius L.) during colour-transition
Source: PeerJ. 2022 Jun 22;10:e13591. doi: 10.7717/peerj.13591 (PMC9233481; doi:10.7717/peerj.13591)

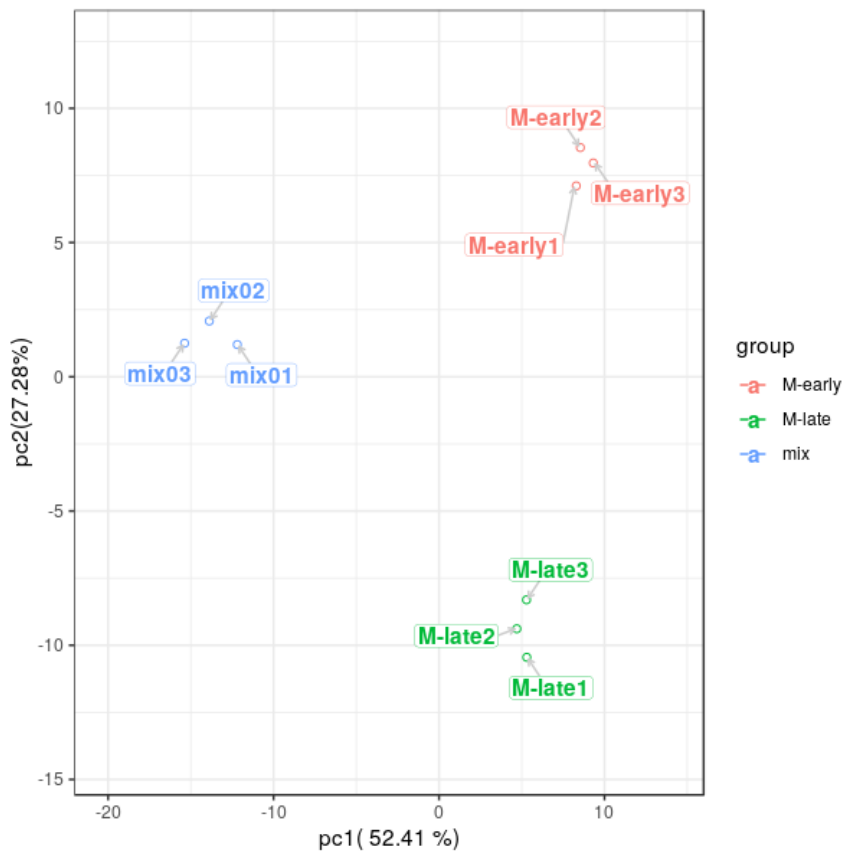

Supplement: Supplemental Information 11 [file peerj-10-13591-s011.pdf]

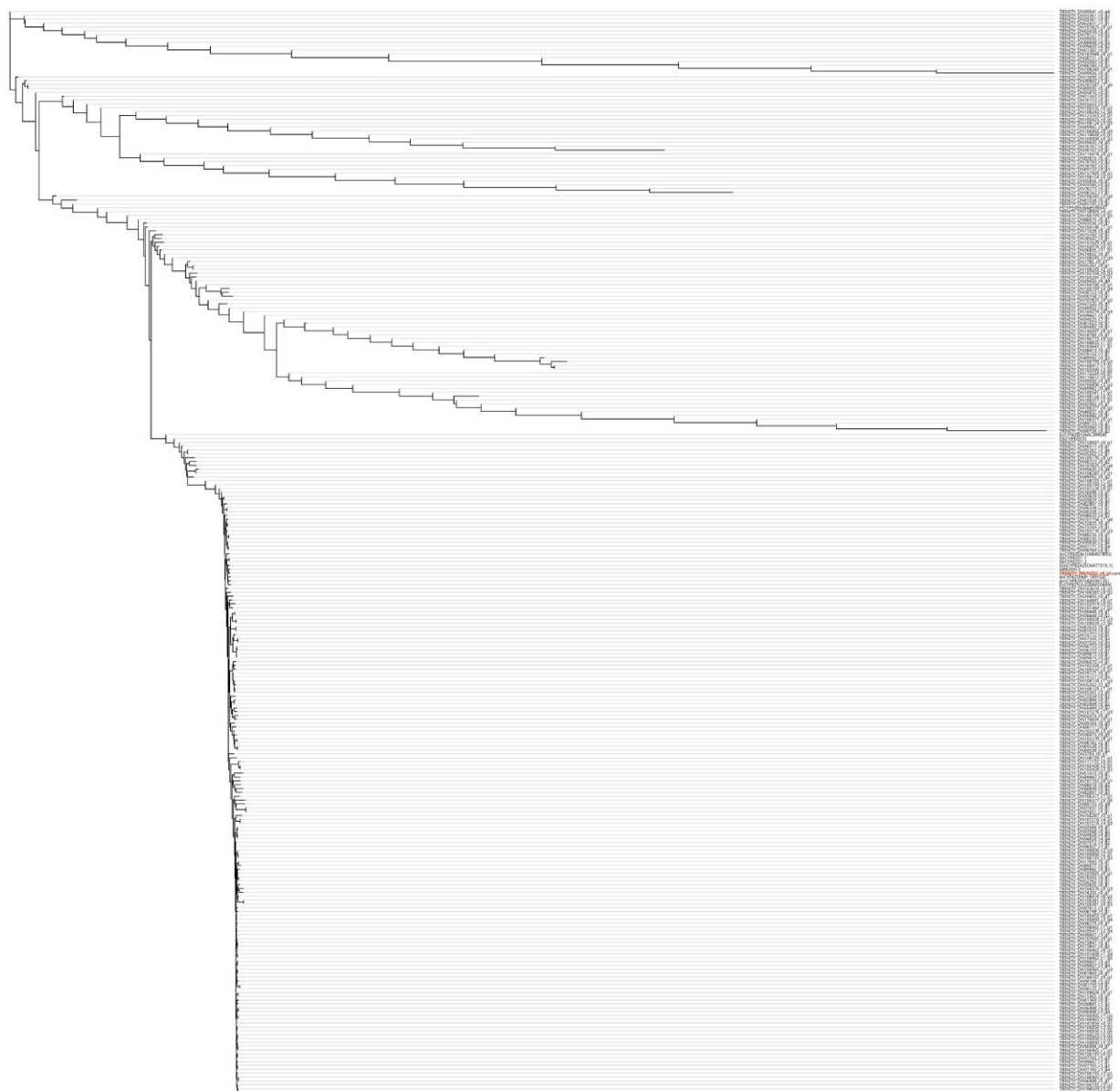

Supplement: Supplemental Information 13 — The candidate genes are shown in red font. [file peerj-10-13591-s013.pdf]

## FLAVONOID BIOSYNTHESIS

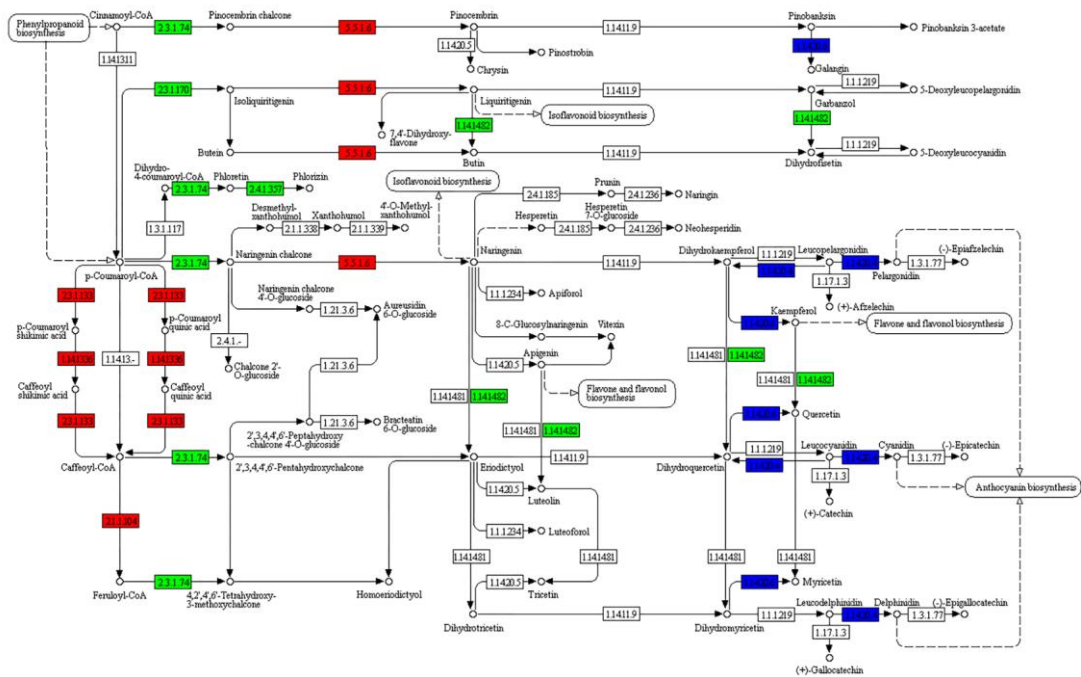

Supplement: Supplemental Information 14 [file peerj-10-13591-s014.pdf]

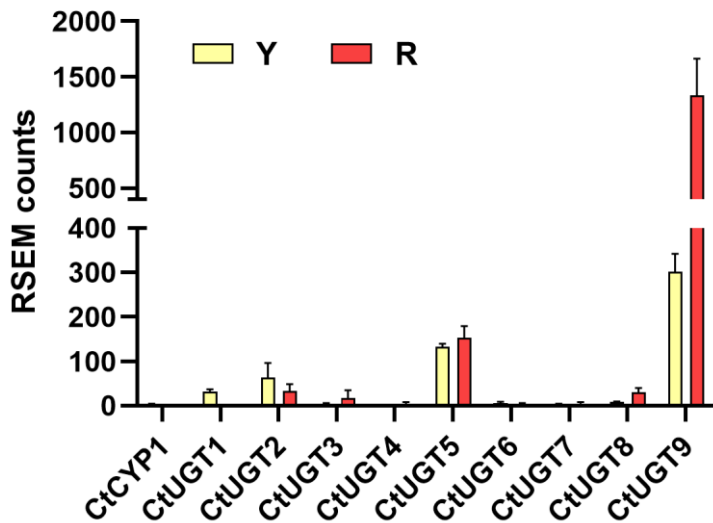

Supplement: Supplemental Information 15 [file peerj-10-13591-s015.pdf]
