# Supplementary material for: Integrated metabolomics and transcriptome analysis on flavonoid biosynthesis in flowers of safflower (Carthamus tinctorius L.) during colour-transition
Source: PeerJ. 2022 Jun 22;10:e13591. doi: 10.7717/peerj.13591 (PMC9233481; doi:10.7717/peerj.13591)
Supplement: Supplemental Information 12 — The candidate genes are shown in red font. [file peerj-10-13591-s012.pdf]

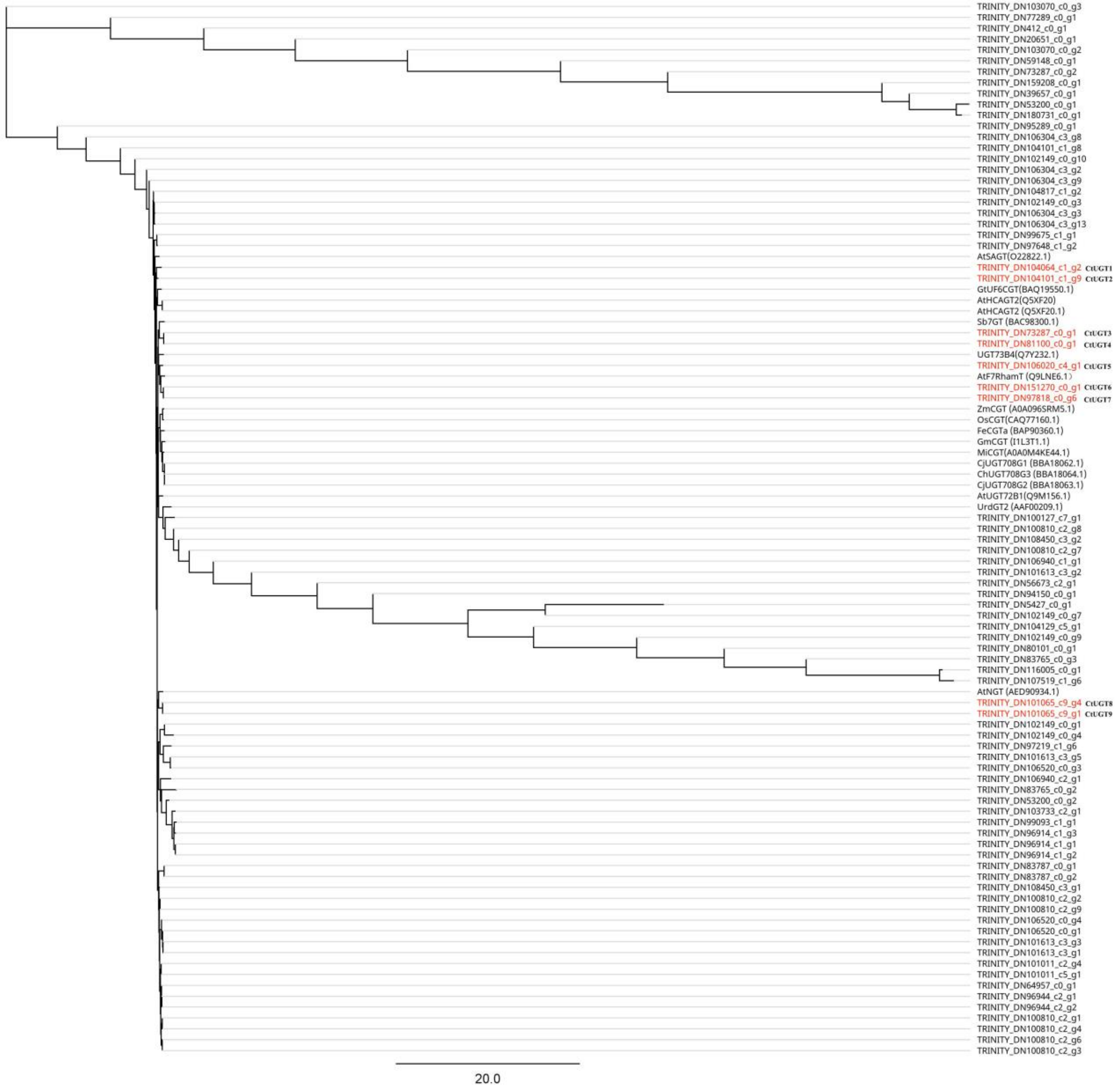
TRINITY_DN103070_c0_g3
TRINITY_DN77289_c0_g1
TRINITY_DN412_c0_g1
TRINITY_DN20651_c0_g1
TRINITY_DN103070_c0_g2
TRINITY_DN59148_c0_g1
TRINITY_DN73287_c0_g2
TRINITY_DN159208_c0_g1
TRINITY_DN39657_c0_g1
TRINITY_DN53200_c0_g1
TRINITY_DN180731_c0_g1
TRINITY_DN95289_c0_g1
TRINITY_DN106304_c3_g8
TRINITY_DN104101_c1_g8
TRINITY_DN102149_c0_g10
TRINITY_DN106304_c3_g2
TRINITY_DN106304_c3_g9
TRINITY_DN104817_c1_g2
TRINITY_DN102149_c0_g3
TRINITY_DN106304_c3_g3
TRINITY_DN106304_c3_g13
TRINITY_DN99675_c1_g1
TRINITY_DN97648_c1_g2
AtSAGT(O22822.1)
TRINITY_DN104064_c1_g2 CtUGT1
TRINITY_DN104101_c1_g9 CtUGT2
GtUF6CGT(BAQ19550.1)
AtHCAGT2(Q5XF20)
AtHCAGT2 (Q5XF20.1)
Sb7GT (BAC98300.1)
TRINITY_DN73287_c0_g1 CtUGT3
TRINITY_DN81100_c0_g1 CtUGT4
UGT73B4(Q7Y232.1)
TRINITY_DN106020_c4_g1 CtUGT5
AtF7RhamT (Q9LNE6.1)
TRINITY_DN151270_c0_g1 CtUGT6
TRINITY_DN97818_c0_g6 CtUGT7
ZmCGT (A0A096SRM5.1)
OsCGT(CAQ77160.1)
FeCGTa (BAP90360.1)
GmCGT (I1L3T1.1)
MiCGT(A0A0M4KE44.1)
CjUGT708G1 (BBA18062.1)
ChUGT708G3 (BBA18064.1)
CjUGT708G2 (BBA18063.1)
AtUGT72B1(Q9M156.1)
UrdGT2 (AAF00209.1)
TRINITY_DN100127_c7_g1
TRINITY_DN100810_c2_g8
TRINITY_DN108450_c3_g2
TRINITY_DN100810_c2_g7
TRINITY_DN106940_c1_g1
TRINITY_DN101613_c3_g2
TRINITY_DN56673_c2_g1
TRINITY_DN94150_c0_g1
TRINITY_DN5427_c0_g1
TRINITY_DN102149_c0_g7
TRINITY_DN104129_c5_g1
TRINITY_DN102149_c0_g9
TRINITY_DN80101_c0_g1
TRINITY_DN83765_c0_g3
TRINITY_DN116005_c0_g1
TRINITY_DN107519_c1_g6
AtNGT (AED90934.1)
TRINITY_DN101065_c9_g4 CtUGT8
TRINITY_DN101065_c9_g1 CtUGT9
TRINITY_DN102149_c0_g1
TRINITY_DN102149_c0_g4
TRINITY_DN97219_c1_g6
TRINITY_DN101613_c3_g5
TRINITY_DN106520_c0_g3
TRINITY_DN106940_c2_g1
TRINITY_DN83765_c0_g2
TRINITY_DN53200_c0_g2
TRINITY_DN103733_c2_g1
TRINITY_DN99093_c1_g1
TRINITY_DN96914_c1_g3
TRINITY_DN96914_c1_g1
TRINITY_DN96914_c1_g2
TRINITY_DN83787_c0_g1
TRINITY_DN83787_c0_g2
TRINITY_DN108450_c3_g1
TRINITY_DN100810_c2_g2
TRINITY_DN100810_c2_g9
TRINITY_DN106520_c0_g4
TRINITY_DN106520_c0_g1
TRINITY_DN101613_c3_g3
TRINITY_DN101613_c3_g1
TRINITY_DN101011_c2_g4
TRINITY_DN101011_c5_g1
TRINITY_DN64957_c0_g1
TRINITY_DN96944_c2_g1
TRINITY_DN96944_c2_g2
TRINITY_DN100810_c2_g1
TRINITY_DN100810_c2_g4
TRINITY_DN100810_c2_g6
TRINITY_DN100810_c2_g3
20.0
